# Nonprofessional Peer Support to Improve Mental Health: Randomized Trial of a Scalable Web-Based Peer Counseling Course

**DOI:** 10.2196/17164

**Published:** 2020-09-21

**Authors:** Samantha L Bernecker, Joseph Jay Williams, Norian A Caporale-Berkowitz, Akash R Wasil, Michael J Constantino

**Affiliations:** 1 Department of Health Care Policy Harvard Medical School Boston, MA United States; 2 Department of Computer Science University of Toronto Toronto, ON Canada; 3 Department of Educational Psychology University of Texas at Austin Austin, TX United States; 4 Department of Psychology University of Pennsylvania Philadelphia, PA United States; 5 Department of Psychological and Brain Sciences University of Massachusetts Amherst Amherst, MA United States

**Keywords:** online learning, nonprofessional education, educational technology, computer-assisted instruction, social support, mental health, psychological stress, eHealth, internet

## Abstract

**Background:**

Millions of people worldwide are underserved by the mental health care system. Indeed, most mental health problems go untreated, often because of resource constraints (eg, limited provider availability and cost) or lack of interest or faith in professional help. Furthermore, subclinical symptoms and chronic stress in the absence of a mental illness diagnosis often go unaddressed, despite their substantial health impact. Innovative and scalable treatment delivery methods are needed to supplement traditional therapies to fill these gaps in the mental health care system.

**Objective:**

This study aims to investigate whether a self-guided web-based course can teach pairs of nonprofessional peers to deliver psychological support to each other.

**Methods:**

In this experimental study, a community sample of 30 dyads (60 participants, mostly friends), many of whom presented with mild to moderate psychological distress, were recruited to complete a web-based counseling skills course. Dyads were randomized to either immediate or delayed access to training. Before and after training, dyads were recorded taking turns discussing stressors. Participants’ skills in the *helper* role were assessed before and after taking the course: the first author and a team of trained research assistants coded recordings for the presence of specific counseling behaviors. When in the *client* role, participants rated the session on helpfulness in resolving their stressors and supportiveness of their peers. We hypothesized that participants would increase the use of skills taught by the course and decrease the use of skills discouraged by the course, would increase their overall adherence to the guidelines taught in the course, and would perceive posttraining counseling sessions as more helpful and their peers as more supportive.

**Results:**

The course had large effects on most helper-role speech behaviors: helpers decreased total speaking time, used more restatements, made fewer efforts to influence the speaker, and decreased self-focused and off-topic utterances (*d*s=0.8-1.6). When rating the portion of the session in which they served as clients, participants indicated that they made more progress in addressing their stressors during posttraining counseling sessions compared with pretraining sessions (*d*=1.1), but they did not report substantive changes in feelings of closeness and supportiveness of their peers (*d*=0.3).

**Conclusions:**

The results provide proof of concept that nonprofessionals can learn basic counseling skills from a scalable web-based course. The course serves as a promising model for the development of web-based counseling skills training, which could provide accessible mental health support to some of those underserved by traditional psychotherapy.

## Introduction

### Background

The mental health care system in the United States fails to meet the needs of millions of people, prompting numerous calls for *disruptive innovations* in mental health care delivery [1,2]. Several gaps in the current system point to the need for such innovations. First, many people with mental illness are unable to access treatment; the number of people with mental illness far outstrips available resources, and cost and other structural barriers are pervasive [3,4]. Second, others choose not to seek help because of negative beliefs about treatment [5,6]. Finally, the mental health system is not designed to address the adverse effects of subclinical symptoms and chronic stress that affect even those without diagnosable mental illnesses [7], and which increases the risk of future psychological and physical decline [8].

Self-guided digital technologies, including self-help apps and chatbots, have been proposed as solutions because of the advantages they provide in access and cost, but they are not a panacea, displaying several limitations [9,10]. Their reach is limited because people seeking mental health support typically prefer face-to-face over computerized therapy [11,12]. Their efficacy is limited because digital tools often fail to motivate and engage users [13,14]. They also currently lack the human-level intelligence required to address nuanced problems [15,16]. It appears that until realistic artificial intelligence is available, many people require human-delivered interventions to meet their preferences, engage them, and respond to their unique concerns. However, this raises the question of how human-delivered interventions could solve the problems with traditional treatments that digital interventions have been created to address—how can human-delivered interventions scale to reach an enormous number of people with mental illnesses, appeal to those who are not interested in professional care, and reduce the burden of subclinical symptoms and stress?

We propose one possibility for a human-delivered solution to address these needs: a *Crowdsourcing Mental Health* (CMH) model that leverages the benefits of technology to overcome treatment barriers while addressing limitations of technology by incorporating the important human element. In the proposed model, digital tools could be used to train nonprofessionals, who would then counsel their peers face-to-face. Even if it is less potent than traditional psychotherapy, such a scalable intervention could have considerable public health impact because of its greater reach [17], a possibility corroborated by survey research. In a survey of more than 500 internet users, 64% of respondents indicated that they would participate in reciprocal peer counseling using skills that they and a peer learned via a web-based course [18]. More than 50% of the respondents who stated that they would never seek psychotherapy or medication expressed willingness to try this model—an important indicator that some of those underserved by traditional treatments could benefit from reciprocal peer counseling.

### Design Considerations for a Peer Counseling Program

We propose 3 features to include in the design of a nonprofessional peer counseling program if it is to meet the aforementioned gaps in traditional mental health care by scaling to meet demand, appealing to those who do not want to seek professional care, and treating subclinical symptoms and stress, all while incorporating human interaction. These features are (1) transdiagnostic applicability (ie, applicability regardless of diagnosis), (2) reciprocity between peers, and (3) scalability of training. In this section, we highlight the relevant literature from which these design considerations were derived. We discuss how these features can address the above gaps and provide additional benefits, and we describe how these features might be implemented.

#### Transdiagnostic Applicability

Applicability to a wide range of problems provides several advantages for nonprofessional peer counseling interventions. This could increase the appeal of the intervention to those who are reluctant to see a professional: if the intervention is appropriate regardless of whether one has received a diagnosis, participants would not need to identify themselves as having a mental illness or to see their symptoms as “severe enough” to merit professional treatment, which are among the most common reasons individuals choose not to seek care [5,6]. In addition, a broad intervention could address the growing number of individuals with impairing subclinical symptoms or chronic stress [7], which increases the risk for mental and physical health problems [8] in addition to the direct distress they cause. Finally, a domain-general intervention can be useful if individuals cannot receive accurate diagnosis (which is challenging in the absence of a professional) [19]; a simpler screening for the level of severity or appropriateness of peer counseling may be viable.

How might this transdiagnostic applicability be achieved? Among extant transdiagnostic treatments, supportive psychotherapy may be especially well-suited for peers with limited mental health training. In supportive psychotherapy, the therapist does not target a specific symptom but rather follows the support seeker’s lead while providing “reflection, empathic listening, encouragement, and [an opportunity] to explore and express ... experiences and emotions” [20]. Thus, support seekers can address whatever problems may arise, including psychological symptoms and “normal” stressors [21]. These techniques align well with what support seekers desire from nonprofessional social support, making supportive psychotherapy especially appropriate for peer delivery [22,23]. Furthermore, in contrast with many transdiagnostic treatments that require extensive training and supervision to implement with fidelity [24,25], supportive psychotherapy’s abbreviated list of specific techniques may render it easier to learn, although this is ultimately an empirical question.

There is consistent evidence that supportive psychotherapy improves psychological symptoms. For example, in randomized controlled trials for depression, it has medium effects versus wait-list or no treatment (approximate *d*=0.6) [20,26]. Compared with treatments that directly target the symptoms or theorized root causes of a particular disorder, supportive psychotherapy does appear at a small disadvantage, with relative effects around *d*=−0.2, but there is some indication that this difference could be driven partly by publication bias or unequal dosages [20,27,28]. Indeed, several meta-analyses have failed to find differences between supportive psychotherapy and gold-standard cognitive behavioral therapy for generalized anxiety disorder [29,30]. In addition, several randomized trials have found no or minimal differences between supportive psychotherapy and directive or expressive treatments for a variety of other conditions, including borderline personality disorder [31], posttraumatic stress disorder [32], social anxiety disorder [33], generalized anxiety disorder [34], anorexia nervosa [35], personality disorders characterized by fearful behaviors [36], and comorbid chronic depression with alcohol dependence [37]. Consequently, some have argued that supportive psychotherapy should be regarded as a “therapy of choice” rather than a control condition [36,38].

This is not to deny that, in many cases, specific techniques that target symptoms or causes may increase the potency of treatment or may be necessary to achieve remission; for example, there is an increasing consensus that treatments that incorporate exposure are superior for anxiety disorders [39-41]. However, even if less powerful than such disorder-specific treatments, delivery of supportive psychotherapy skills on a large scale by laypeople could have a substantial public health impact, especially in cases where the alternative to supportive peer counseling is no treatment at all [17]. Determining the appropriate population for peer-delivered supportive psychotherapy techniques should be guided by future clinical trials, but we propose that this intervention may be a strong fit for any individual with subclinical distress and prodromal symptoms as well as for individuals with mild to moderate mental illness across a spectrum of disorders (eg, anxiety and related disorders, mood disorders, substance use disorders, and eating disorders) who would not otherwise seek treatment.

#### Reciprocity Between Peers

There are many advantages to making supportive psychotherapy delivered by nonprofessionals reciprocal, such that 2 members of a dyad both give and receive support, as opposed to unidirectional, such that one member takes on a patient role and the other a counselor role. A reciprocal model has a major advantage in scaling to meet demand. Unidirectional solutions such as task shifting to trained nonprofessionals would require a multiple-thousands-fold increase in employees delivering therapeutic services full time to treat all individuals with mental health difficulties [42,43]; in contrast, a reciprocal model does not demand a large change in the workforce. Instead, reciprocal peer counseling requires only a few hours of each person’s leisure time, and each person is compensated via (1) receiving support in return and (2) the benefits of providing support to others. In a sense, this model crowdsources mental health care by dividing the enormous undertaking of treating mental illness into manageable tasks carried out by laypeople.

Reciprocal peer counseling may also appeal to those who would not seek professional assistance (or, indeed, request it from their friends) because of the threat to self-esteem associated with being a person who needs help or because of concerns about burdening others. Indeed, unidirectional support receipt is sometimes associated with negative mood, potentially because of these features [44]. In contrast, reciprocity of support maintains an egalitarian relationship, and the opportunity to act as a support provider can protect health and improve mood [45,46], in some cases even more than receiving support [47].

The involvement of a peer can also remedy a limitation of most web-based self-help programs, that is, nonadherence or withdrawal from the program. A recent meta-analysis of clinical trials of smartphone apps for treating anxiety and depression found that 26% of participants withdrew (closer to half when adjusting for publication bias); however, the inclusion of human interaction reduced dropout to close to 12% [48]. Interaction with another person can provide a sense of accountability [49,50]; indeed, in a reciprocal program, participants might be especially motivated to persist because in addition to promoting their own well-being, they know another person is benefitting from their involvement.

Finally, a reciprocal peer counseling model may attack a driver of psychological ill health at its root. The detrimental health and mortality effects of social isolation and loneliness are well established [51,52], and perceived social support protects against mental illnesses [53-55]. Reciprocal self-disclosure generates intimacy [56], so taking turns as helper and client could increase perceived social support in a manner that is not present in traditional psychotherapy. Thus, a peer counseling program using this format could improve psychological well-being through 2 classes of mechanisms: it could not only give participants an opportunity to address the sources of their distress but could also generate feelings of closeness and support.

#### Scalability of Training

To meaningfully address mental health care shortages, training for peer counselors must be widely accessible at scale. To achieve the required reach, training should have little or no monetary cost, should be available regardless of geographic location or population density, and should effectively train people with varying backgrounds and abilities.

Therefore, we suggest that the training should be available on the web in a self-directed format (although this does limit its use to individuals who have access to an internet-connected device; additional solutions are needed for those who lack such access). Crucially, to reach the required scale, web-based training should be primarily self-guided, rather than requiring a live instructor [57]. Otherwise, the number of human trainers available would act as the limiting factor in the number of people who could be served, and human involvement would drive costs. This approach is consistent with the evolution of massive open online courses, which are increasingly taught in a self-paced format. However, it is far from guaranteed that a self-guided web-based course could effectively teach interpersonal skills, especially to nonprofessionals who may be experiencing psychological symptoms; as discussed below, the literature on web-based interpersonal skills training is limited. Consequently, such a course must be carefully designed, drawing on the science of learning and research on online pedagogy.

### Research on Extant Web-Based Therapeutic Skills Training Programs

In this section, we briefly review the supporting evidence for web-based programs that have been created to teach related skills, and we explain how the proposed intervention differs from that work.

One group of existing web-based training programs includes courses for professionals in evidence-based psychotherapies [58] and distance education programs for graduate-level counselors [59]. These programs have succeeded in increasing knowledge, self-reported skill, and, more rarely, observed skill. However, these differ from the proposed peer counseling course in 2 consequential ways: (1) they often involve considerable instructor and student interaction through telecommunication, making them difficult to expand, and (2) they teach nondistressed groups that have self-selected for aptitude and interest in mental health care delivery [58].

Another handful of web-based peer support platforms train nondistressed volunteer listeners, but unlike our program, these platforms generally do not use evidence-based behavioral teaching techniques, and it is unknown if these trainings improve listeners’ behavioral adherence to guidelines [60-62].

Finally, several web-based romantic relationship enhancement programs exist, some of which teach communication skills, and these often do target couples in distress. However, studies of these programs have only shown benefits for relationship outcomes and have rarely measured changes in interpersonal behaviors [63,64] (refer to the study by Braithwaite and Fincham [65] for an exception).

Across all these types of programs, rather than rigorously evaluating observed behavior, tests of teaching efficacy tend to rely on assessments of learners’ *self-reported* perceived skills and book knowledge, which may be only weakly correlated with a learner’s ability to implement skills in a real interpersonal interaction [57]. We address this limitation in this study.

### This Study

Owing to these gaps in the literature, it remains unclear whether a training program fitting our design desiderata would be effective—in other words, it is unknown whether nonprofessionals, including people reporting moderate psychological distress, could learn peer counseling skills via a self-guided web-based course. To address this question, we developed a peer counseling program called Crowdsourcing Mental Health (CMH). CMH fulfills our design criteria: it is a reciprocal program that begins by teaching pairs of peers supportive psychotherapy skills via a self-guided, web-based course. Thus, it may have the potential to address the current limitations of mental health care systems around access, appeal, and treatment of subclinical symptoms. Of course, CMH and other similar peer counseling programs are far from the sole solution; they are unlikely to be appropriate for some of the most vulnerable or most ill or those who have specific limitations around technology use or peer interactions. However, reciprocal peer support programs can add strong value to a portfolio of novel mental health interventions to fill gaps in the current health care system.

In this study, we describe CMH’s development and report on a randomized trial designed to test its efficacy in improving skill use, adherence to guidelines, and perceived helpfulness by evaluating users’ performance in recorded CMH sessions and their postsession reactions. The primary research questions (RQs) were as follows:

- RQ 1: How much does the course change the use of specific helping skills? By estimating changes in the use of individual skills, we can differentiate skills that were effectively taught from those ineffectively taught, informing revision of specific course sections. We hypothesized that helpers would increase the use of 2 behaviors prescribed by the course, would decrease the use of 2 behaviors proscribed by the course, and would decrease in-session speaking time. (We also measured some common behaviors that were neither prescribed nor proscribed and had no strong hypotheses about changes in those.)
- RQ 2: Does the course improve helpers’ overall adherence in delivering helping skills? We predicted an increase in adherence from pre- to posttraining.

As the primary goal of this study was to investigate the teaching effectiveness of the course and not its impact on mental health, participants were not required to meet with their peers after completing this study; consequently, the mental health effects of repeated peer interactions could not be determined. However, as a proxy measure of whether a reciprocal peer counseling intervention of this kind could produce mental health benefits, we assessed participants’ perceptions of the short-term impact of using the skills during the in-laboratory counseling sessions, enabling us to address the following RQ:

- RQ 3: Does the talker’s perception of session helpfulness increase after taking the course? We hypothesized that talkers would perceive the sessions after training as more productive and that they would feel closer to their peers—in other words, that changes would take place in the 2 proposed mechanisms of reciprocal peer counseling.

## Methods

### Course Design

In CMH, pairs of acquaintances take a web-based course that teaches helping skills, which are the focus of this investigation, as well as talking skills, which consist of guidelines drawn from the literature on coping and emotion regulation. Both participants learn both roles. Once each person has completed the web-based course on his or her own, the peers can then meet for mutual support sessions, taking turns in the helper and talker roles. To address the design consideration of transdiagnostic applicability, CMH’s helping skills parallel the skills of supportive psychotherapy [38], which are also the core skills taught in the dominant counselor training models [66,67]. These skills include taking a warm and nonjudgmental attitude, listening attentively without attempting to influence the speaker, and using techniques to elicit reflection and elaboration (eg, paraphrasing and asking open-ended questions).

The CMH course consisted of 10 lessons, 5 on taking the helper role and 5 on taking the talker role. (The talking skills lessons were included because CMH users may be therapy-naïve and have difficulty directing their own sessions. These lessons gave instructions on how to explore a stressor, describe emotions, and develop a coping plan. As talking performance was not the focus of this study, we do not discuss these lessons further.) Each of the helper lessons addressed 1 of the following 5 topics: focusing one’s attention on the talker, taking an accepting and caring attitude, avoiding unhelpful attempts to influence the talker, restating (paraphrase and summary), and asking open-ended questions.

The success of skills training is dependent on the pedagogical methods used [68]. In some previous studies of web-based psychotherapy skills training, self-guided instruction (which scales more easily) has been found to be inferior to self-guidance plus videoconference role-play with an instructor [69-71]. Owing to the need to address the design consideration of the scalability of training, we considered such use of videoconference to be infeasible for a widely disseminated and low-cost course. Therefore, we carefully developed alternative training strategies, relying on extensive review of basic and applied research on learning and online education to identify ways we could maximize efficacy while minimizing human-delivered instruction.

#### Implementation of Behavior Modeling Training as a Teaching Method

Behavior modeling training (BMT) is the best-supported set of techniques for increasing the performance of interpersonal and other behavioral skills [72], and it has been used effectively to teach nonprofessionals basic counseling and active listening skills in face-to-face settings [73,74]. Consequently, BMT served as the pedagogical foundation for the course. BMT includes 4 components: learners receive a description of each skill (*instruction*); view other people performing skills (*modeling*); practice skills, often through role-play (*practice*); and receive performance feedback (*feedback*).

To make the course scalable, these 4 components needed to be translated into a primarily self-guided, web-based format. Instruction and modeling were relatively simple to implement and took the form of videos: audio instruction was accompanied by text and images, and diverse volunteer actors modeled the skills. In creating these portions of the course, we also drew on training techniques identified through basic and applied research from areas as diverse as knowledge acquisition [75], motor learning [76], and computer-assisted instruction [77].

As noted earlier, practice and feedback are more challenging to translate into a primarily self-guided format. To implement practice, the course simulated interpersonal interactions with increasing degrees of complexity and realism, beginning with lower-fidelity, simpler automated exercises, and progressing toward live interactions. This approach has dual benefits: first, it can scaffold learning rather than immediately forcing learners to juggle the stimuli and challenges of a face-to-face conversation [78], and second, minimizing human involvement improves convenience and scalability. In the CMH course, learners began by typing responses to video-recorded actors, then progressed to practicing 3 times over the phone with a minimally trained mentor, and finally held 3 in-person practice sessions with the peer whom they had selected as their partner in the intervention. The demands of the mentor role were designed to be extremely minimal (eg, reading from a script) so that when CMH is publicly launched, any individual who uses CMH could volunteer to mentor new learners, eliminating the resource limitations associated with requiring trained instructors. For this study, undergraduate research assistants served as the telephone mentors.

Feedback took the form of self-assessments because of the challenges of providing nuanced human-delivered or machine-coded feedback at scale [79,80] and the risk associated with an untrained peer providing inaccurate or anxiety-provoking feedback [81]. After each exercise, the learners answered a series of questions about whether they followed each instruction. By assessing granular behaviors, learners can identify behaviors to change in the future while minimizing the threat to self-esteem and ensuing negative affect that could impede learning [82]. They were not asked to give themselves a global evaluation because self-evaluations are more accurate when specific and objective tasks are assessed [83].

#### Course Development Process

The course content was written by the first author, using BMT as an organizing framework for teaching the set of behavioral skills from supportive psychotherapy. Manuals on teaching counseling skills and motivational interviewing were consulted [66,67,84], both to ensure that no relevant skills were missed and to inform the design of practice exercises. When generating examples for the modeling portion of the course, we attempted to represent individuals with diverse life experiences and demographic characteristics (eg, socioeconomic status, race, and age). The written course content was then reviewed by another clinical psychologist and an online education researcher and was read and pilot tested by 2 research assistants.

After the first round of revisions to the written materials, a digital prototype of the course was created, including creating instructional and modeling videos and interactive exercises. These materials were designed in keeping with research on e-learning [77] to optimize visuals, narration style, and other elements for educational efficacy. The videos were edited using Camtasia software (TechSmith) and hosted on the TechSmith website, which allows for embedding quiz questions within videos. All course materials were hosted on the web using Qualtrics Research Suite survey software, which enabled additional interactive exercises in a variety of formats (eg, multiple choice and short answer questions). This digital version of the course was then pilot tested with 5 volunteers. Final revisions were made based on volunteers’ feedback as well as observations of their performance.

### Participants

#### Inclusion and Exclusion Criteria

We sought a sample with somewhat elevated distress through our recruiting methods (ie, by framing the program as a way to reduce stress), but we did not exclude participants with low distress because we (1) did not want to make it more difficult for participants to find eligible partners and (2) hoped that CMH may be a useful tool for prevention and personal growth even in the absence of current symptoms. Although we expected that this peer counseling model would be appropriate for those with more severe symptoms, we decided to limit initial testing of the course to those with milder distress for safety and ethical reasons. Therefore, we excluded individuals scoring more than 2 SDs above general population norms on the Brief Symptom Inventory (BSI) [85] or responding in the affirmative to the BSI item on suicidal thoughts.

We excluded individuals currently receiving psychotherapy, given that they already have access and willingness to seek care and, therefore, are not in CMH’s highest-priority target population. We did not exclude those taking psychiatric medication because it may be a weaker indicator of access to care (eg, some people might be prescribed medication through a general practitioner without having access to specialist treatment).

Additional eligibility criteria included being aged 18 years or older; having access to an internet-connected computer; and being able to speak, read, and write in English.

#### Sampling and Recruitment Method

Participants were recruited from several medium-sized towns (population 20,000-40,000) in the Western Massachusetts region. The study was advertised via flyers, web-based classifieds, and announcements on listservs and in college student groups. Advertisements presented the program as an opportunity to learn skills to reduce stress and to develop closeness with another person.

Recruitment followed a multistep process in which a first participant was recruited and screened, and then that individual recruited a peer from their existing social network. The first participant was discouraged from selecting first-degree relatives, romantic partners, or individuals with whom their relationships were characterized by conflict or disagreement. However, to increase the external validity of the study, no potential peer pairings were forbidden. Of the 30 initial participants enrolled in the study, 29 (97%) participated with their first-choice peers and 1 (3%) participated with her second-choice peer.

The sample of 60 individuals (30 pairs) comprised adult community members (18/60, 30%) and full-time undergraduate students (42/60, 70%). Of the 60 individuals, 42 (70%) identified as women, 15 (25%) as men, and 3 (5%) as transgender or gender nonconforming. They were aged 18 to 62 (median 20.5) years. The most common racial and ethnic identities were White non-Hispanic or Latinx (35/60, 58%), East Asian (10/60, 17%), and White Hispanic or Latinx (5/60, 8%), with the remainder identifying as South Asian (4/60, 7%), multiracial (3/60, 5%), Black (2/60, 3%), and Native American Hispanic or Latinx (1/60, 2%).

### Measures

#### Psychological Distress

To assess psychological symptoms, we administered the BSI [85]. The BSI is a 53-item measure on which respondents rate symptoms experienced within the past week on 9 mental illness dimensions, from which an index of total distress can be calculated. This measure was chosen because it assesses symptoms of a range of disorders and summarizes them in a single index, has strong psychometric properties, and has published norms for patient and nonpatient populations. In the sample of this study, the BSI showed strong internal consistency in all sessions (coefficient α range=.95-.97).

To assess perceived stress, we administered the 10-item Perceived Stress Scale (PSS-10) [86]. This measure was chosen because although scores are correlated with psychological symptoms, the construct of stress as measured by this scale is distinct from mental illness and predicts future symptoms above and beyond current symptom measures [86]. The PSS-10 has been validated in numerous studies, and published norms also exist. In the sample of this study, internal consistency was good at all time points (coefficient α range=.82-.88).

#### Coding System for Skill Performance

The performance of participants in the helper role was evaluated using a study-specific coding system based on the psychometrically established Helping Skills Scale [87]. This coding system was developed specifically to assess participants’ use of the skills taught in the course (and avoidance of proscribed behaviors).

Conversational turns are segmented into sentence-like grammatical units, and each unit is coded as falling within a certain category. The system is not intended to capture all possible categories of verbal utterances, but instead codifies behaviors that are prescribed or proscribed in the CMH course or that are very common in social support interactions. The coding system includes 6 mutually exclusive categories: *restatement* and *open-ended question* (central CMH skills), *closed-ended question* (discouraged by the course), *self-disclosure* and *sympathy* (common response modes that are neither prescribed nor explicitly proscribed, although we regarded excessive self-disclosure as evidence of failure to focus on the talker), and *other*. The system also includes a nonmutually exclusive category called *influencing*. Any speech unit in which the helper attempts to problem solve or change the talker’s emotional response (which is proscribed by the course) is coded as influencing, in addition to its classification in 1 of the 6 primary categories. The 8 outcome variables for RQ 1 were the total number of sentence units uttered and the proportion of speech units in each category (the 6 mutually exclusive categories plus influencing).

Although these proportions provide a detailed profile of how helper behaviors change, they do not reveal whether learners increase their overall adherence to the guidelines given in the course. Therefore, to address RQ 2, we created a composite index of adherence derived from the coded speech units. Participants are awarded points for engaging in behaviors encouraged by the course and are docked points for proscribed behaviors (eg, they earn points if restatements form a high proportion of the session; points are subtracted depending on the number of units of advice giving). This scale has a theoretical range of −50 to +25.

The coding system was applied by the first author, who developed the system, and a team of 9 trained undergraduate research assistants, all of whom were blinded to session condition and time point. Psychometrics and training procedures are reported in Multimedia Appendix 1 [88-94].

#### Perceived Session Helpfulness

To address RQ 3, both participants in each dyad rated how helpful the sessions were to them using the Crowdsourcing Mental Health Session Reaction Scale (CSRS; see Multimedia Appendix 2 for the instrument), a modified version of the Revised Session Reaction Scale [88] that focuses on their experiences when they were in the talker role. The CSRS items loaded on 2 subscales: task reactions (6 items), which reflect progress toward the resolution of the problem through insight, emotional relief, or problem solving, and relationship reactions (3 items), which reflect feeling understood by, connected to, and supported by one’s peer. Thus, this measure addresses both types of potential mechanisms of peer counseling: resolution of distress and increased perceptions of closeness and support. Both subscales have a theoretical range of 1 to 9. Internal consistency of each subscale was good at all laboratory visits (coefficient of α range=.86-.92 for task reactions and .84-.95 for relationship reactions). Multimedia Appendix 1 provides scale development details.

### Procedure

All study procedures were approved by the University of Massachusetts Amherst institutional review board.

#### Study Design

**Figure 1:**
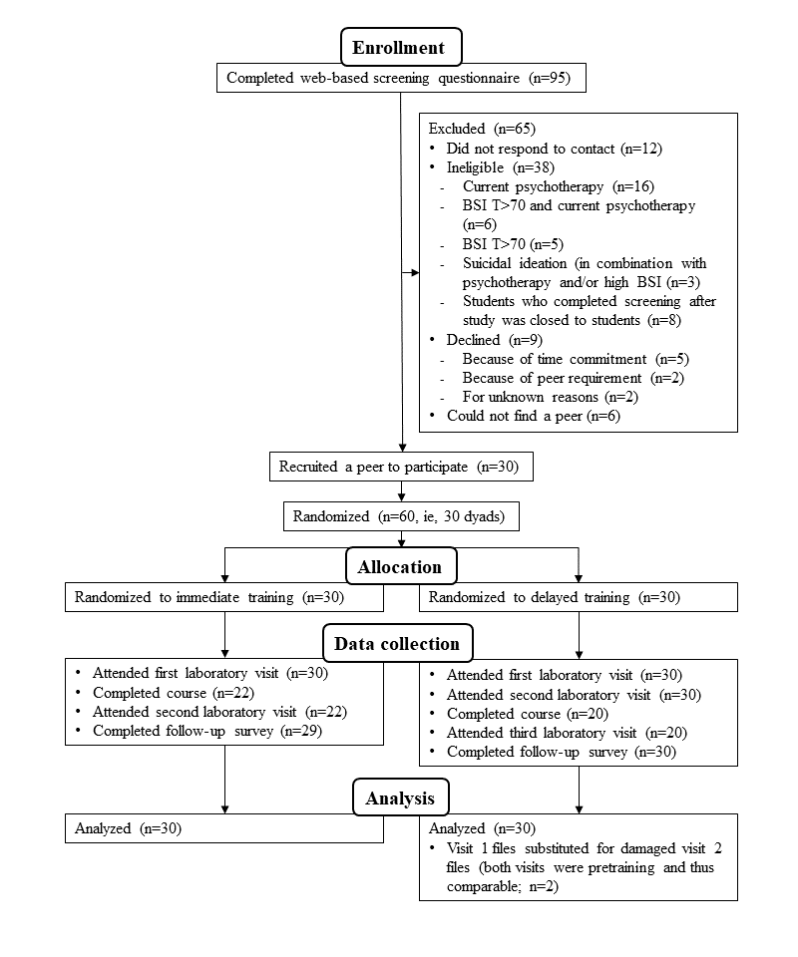
Study flow diagram. N denotes the total number of individuals, not the number of dyads. BSI: Brief Symptom Inventory.

This randomized experiment used a pretest-posttest wait-list controlled design to assess whether participants’ behavior changed because of taking the course. Half of the dyads were randomized to an immediate training condition and half to a wait-list control (ie, delayed training) condition using a random number generator. The dyads in the immediate training condition were recorded while discussing stressors before and after completing the course over a 4-week period, whereas the dyads in the delayed training condition engaged in 2 stressor discussions separated by 4 weeks, then took the course for 4 weeks, and ultimately completed a final stressor discussion. Participants in both conditions were contacted weekly to address any questions or concerns. Figure 1 depicts the participants’ flow through the study.

The randomized wait-list controlled element of this study design enabled us to determine whether behavioral changes could be causally attributed to the course by evaluating between-group differences in behavior change from the first to second laboratory visit. Collecting data on *all* participants’ pre- and postcourse behavior allowed us to obtain a more precise estimate of the magnitude of behavior change by analyzing data from all participants in a pre-post design.

#### Stressor Discussions

Stressor discussions were administered by trained research assistants according to a script and took place in treatment rooms in the university’s clinical psychology training clinic, which provided an intimate, comfortable setting along with a means for nonintrusive video recording. At each laboratory visit, participants took turns talking and listening about stressors, taking 30 min each in the talker and helper roles. The order of turn-taking was determined by a coin flip. A careful procedure for selecting stressors was used so that the severity of stressors was comparable across laboratory sessions: at visit 1, participants named 3 current stressors, rated their severity, and chose the second-most severe stressor; at subsequent visits, participants named 3 current stressors they had not previously discussed, rated their severity, and chose the stressor closest in severity to the stressor discussed previously.

In the precourse sessions, participants were told to disclose and respond as they would naturally. After taking the course, they were told to talk and respond using the skills they learned in the course; the instructions specified that they should use the skills “as they would when meeting outside of the lab rather than trying to impress anyone” to maximize ecological validity and reduce experimenter demand.

Participants were compensated for their time after each laboratory visit, US $50 for the precourse visits and US $70 for the postcourse visit. To minimize the impact of compensation on motivation to learn, the payment scheme was explained using language intended to encourage participants to construe payment as compensation for their laboratory visits, not for taking the course.

### Data Analysis

All data analyses were planned a priori*.* We estimated 2 models to test the effects of the course on each outcome variable. First, to estimate the within-subjects magnitude of change from pre- to posttraining, we aggregated the pretraining and posttraining visits across conditions and tested the effect of time. Second, to establish whether changes could be attributed to the training (as opposed to, eg, repeated testing, maturation, and similar threats to internal validity) through a between-subjects analysis, we examined only the first 2 visits, testing the effects of time, condition, and their interaction to assess whether change from visit 1 to visit 2 was greater in the immediate training condition than in the delayed or wait-list condition. We used multilevel modeling to account for the nesting of time points within persons and the nesting of persons within dyads. As the limited number of data points would make such models unidentified, precluding maximum likelihood or related methods [95], we used Bayesian data analysis in the R package *brms* [89]. Bayesian data analysis produces a posterior distribution for each parameter that indicates the relative probability of all possible values in light of (1) the observed data and (2) a prior distribution that represents the possible values of the parameters as known or believed before data collection. Parameter estimates are typically summarized by the central tendency of the posterior (eg, the mean of the distribution) and the 95% credibility interval (CI), which is the range that contains 95% of the probability density of the posterior. When the 95% CI excludes 0 (or 1 in the case of odds ratios), one can conclude that an effect likely exists in the population. For all models, conservative priors were chosen, such that posterior distributions were influenced almost exclusively by the data. Unless explicitly stated otherwise, all analyses were planned a priori. More details, including model equations, are given in Multimedia Appendix 1.

## Results

### Participant Characteristics

Demographic and baseline clinical characteristics of the sample are provided in Table 1. The average participant’s global psychological symptoms fell 1 SD above the general population (nonpatient) norms, indicating that a substantial proportion of participants were experiencing elevated distress.

Participants reported a variety of relationship types. Most (17/30, 57%) pairs were friends; 20% (6/30) of pairs were in a romantic relationship, 10% (3/30) of pairs were coworkers, 10% (3/30) of pairs were roommates, and 3% (1/30) of pairs consisted of a mother and daughter. One-third of the pairs had been acquainted for less than 1 year, and one-fourth of the pairs had known each other for more than 10 years (median relationship length=2.5 years).

**Table 1:** Baseline demographic and clinical characteristics.

Characteristics		Values
**Age (years; n=60)**		
	Mean (SD)	24.6 (12.4)
	Median	20.5
**Gender (n=60), n (%)**		
	Woman	42 (70)
	Man	15 (25)
	Transman	2 (3)
	Genderqueer woman	1 (2)
**Race and ethnicity (n=60), n (%)**		
	White non-Hispanic or Latinx	35 (58)
	East Asian	10 (17)
	White Hispanic or Latinx	5 (8)
	South Asian	4 (7)
	Black	2 (3)
	Native American, Hispanic or Latinx	1 (2)
	Multiracial	3 (5)
Born outside the United States (n=60), n (%)		13 (22)
Nonnative English speakers (n=60), n (%)		8 (13)
**Educational level (n=60), n (%)**		
	Some college education	41 (68)
	Associate’s or technical degree	1 (2)
	Bachelor’s degree	9 (15)
	Some graduate or professional school	3 (5)
	Graduate or professional degree	6 (10)
**Marital status (n=60), n (%)**		
	Never married	51 (85)
	Married	7 (12)
	Separated or divorced	2 (3)
**Household income (n=55), US** $		
	Mean (SD)	96,000 (86,000)
	Median	80,000
**Income/√ household members^a^ (n=55), US** $		
	Mean (SD)	54,000 (43,000)
	Median	42,000
**Visit 1** **Brief Symptom Inventory** **T score^b^ (n=60)**		
	Mean (SD)	60.8 (9.3)
	Median	61.5
**Visit 1** **Perceived Stress Scale-10** **(n=60)**		
	Mean (SD)	18.7 (5.8)
	Median	18
Ever in psychotherapy (n=60), n (%)		19 (32)
**Months in psychotherapy^c^ (n=19)**		
	Mean (SD)	25.8 (31.7)
	Median	10
Would consider psychotherapy (n=60), n (%)		52 (87)
Ever on psychiatric medication (n=60), n (%)		11 (18)
**Months on psychiatric medication^c^ (n=11)**		
	Mean (SD)	41.0 (52.4)
	Median	18
Currently on psychiatric medication (n=60), n (%)		9 (15)
Would consider psychiatric medication (n=60), n (%)		44 (73)

^a^Income/√ household members is included to adjust total income for household size while accounting for economies of scale.

^b^In psychometrics, the T score refers to a normatively adjusted score with a mean of 50 and an SD of 10 (not to be confused with the *t* test statistic).

^c^Refers to total months of treatment over the course of the lifetime; these months were not necessarily one contiguous course of treatment.

### Effects of the Course on Behaviors and Perceived Helpfulness

Means and SDs for each outcome variable are provided in Table 2. Effect sizes presented in the text represent within-person pre-post training changes. Table 3 displays the estimates for the coefficient of interest for each type of multilevel model. For the first model, the coefficient represents the change from pretraining to posttraining among all participants. For the second model, the coefficient represents the degree to which the change from visit 1 to visit 2 was greater in the immediate training condition than in the delayed training condition. When the 95% CI for this coefficient excludes 0 (or 1 for odds ratios), the change can be attributed to the training. The results for all other fixed and random effects are given in Multimedia Appendix 1.

**Table 2:** Descriptive statistics for outcome variables by visit and condition.

Outcome variable		Visit 1^a^, mean (SD)	Visit 2^b^, mean (SD)	Visit 3^c^, mean (SD)
**Total sentence units uttered**				
	Immediate training condition	239.3 (103.0)	112.6 (62.1)	—^d^
	Delayed training condition	252.9 (113.6)	241.5 (77.7)	88.9 (64.2)
**Percent restatement**				
	Immediate training condition	3.0 (3.9)	27.9 (25.8)	—
	Delayed training condition	3.5 (3.2)	2.2 (4.0)	21.9 (17.4)
**Percent influencing**				
	Immediate training condition	30.7 (17.6)	4.8 (6.8)	—
	Delayed training condition	40.5 (21.3)	39.7 (17.0)	12.4 (18.2)
**Percent open-ended questions**				
	Immediate training condition	2.9 (2.1)	6.1 (6.4)	—
	Delayed training condition	2.1 (2.4)	3.7 (3.8)	12.7 (7.7)
**Percent closed-ended questions**				
	Immediate training condition	16.1 (9.6)	13.9 (10.5)	—
	Delayed training condition	9.1 (6.1)	10.9 (7.0)	18.1 (13.1)
**Percent self-disclosure**				
	Immediate training condition	16.6 (13.3)	3.5 (8.2)	—
	Delayed training condition	18.3 (13.1)	19.0 (14.9)	5.2 (9.6)
**Percent sympathy**				
	Immediate training condition	16.1 (12.1)	15.3 (14.3)	—
	Delayed training condition	13.4 (11.5)	12 (9.2)	8.1 (7.7)
**Percent other**				
	Immediate training condition	45.4 (11.8)	33.4 (20.1)	—
	Delayed training condition	53.5 (17)	52.3 (17.7)	34.1 (17.9)
**Adherence score**				
	Immediate training condition	−17.8 (6.7)	4.1 (8.3)	—
	Delayed training condition	−21.3 (8.3)	−22.8 (6.5)	0.9 (12.0)
**CSRS^e^ task reactions**				
	Immediate training condition	5.4 (1.5)	7.0 (1.5)	—
	Delayed training condition	5.4 (1.5)	5.4 (1.5)	7.0 (1.6)
**CSRS relationship reactions**				
	Immediate training condition	7.0 (1.4)	7.1 (1.8)	—
	Delayed training condition	6.6 (1.6)	6.6 (1.6)	7.2 (1.9)

^a^n=60 at visit 1.

^b^n=22 for immediate training and n=28 for delayed training at visit 2.

^c^n=20 at visit 3 (delayed training condition only).

^d^Cells for the immediate training group are blank for visit 3 because the immediate training group only participated in two laboratory visits (see “Study Design” section).

^e^CSRS: Crowdsourcing Mental Health Session Reaction Scale.

**Table 3:** Results of Bayesian multilevel models estimating change from pretraining to posttraining among all participants (model 1) and the difference between the 2 conditions in change from the first to second laboratory visits (model 2).

Outcome variable		Pre-post training effect (model 1), estimate^a^	Pre-post training effect (model 1), 95% CI^b^	Visit×condition interaction (model 2), estimate	Visit×condition interaction (model 2), 95% CI

Total sentence units uttered^c^		−1.88	−2.21 to −1.54	−1.97	−2.47 to −1.46
**Behavior categories^d^**					
	Restatement	16.44	7.92 to 34.81	28.50	6.89 to 119.1
	Influencing	0.09	0.04 to 0.16	0.14	0.07 to 0.28
	Open-ended questions	3.63	2.46 to 5.42	1.40	0.59 to 3.22
	Closed-ended questions	1.15	0.84 to 1.55	0.63	0.36 to 1.07
	Self-disclosure	0.05	0.01 to 0.16	0.09	0.03 to 0.25
	Sympathy	0.86	0.59 to 1.23	1.08	0.59 to 1.95
	Other	0.51	0.36 to 0.71	0.64	0.36 to 1.13
Adherence score^e^		1.64	1.39 to 1.9	1.90	1.48 to 2.32
**Perceived session helpfulness^e^**					
	CSRS^f^ task reactions	0.96	0.69 to 1.26	1.00	0.41 to 1.56
	CSRS relationship reactions	0.28	−0.06 to 0.62	0.20	−0.38 to 0.77

^a^Estimate: mean of estimated posterior distribution.

^b^95% CI: 95% credibility interval.

^c^Count variable with Poisson link function; coefficients are in log units.

^d^Binomial or Bernoulli distributed variables with logistic link function; coefficients are in odds ratio units. For model 1, the estimate represents the relative odds of the behavior at posttraining compared with pretraining; for model 2, the estimate represents the degree to which the relative odds of a behavior at visit 2 compared with visit 1 were higher or lower in the group that underwent immediate training.

^e^Metric variables with identity link function; coefficients are in standardized units (ie, SDs).

^f^CSRS: Crowdsourcing Mental Health Session Reaction Scale.

#### RQ 1: How Much Does the Course Change the Use of Specific Helping Skills?

At baseline, participants’ behaviors were typical of untrained supportive conversations. We observed signs of positive intentions and a lack of hostility (eg, criticism was rare, and advice and encouragement were common). However, helpers did not spontaneously display several other behaviors recommended by supportive psychotherapy guidelines. For example, participants spent more than one-third (34%) of the pretraining session, on average, trying to influence the talker through advice giving and related behaviors, and they delivered relatively few restatements or open-ended questions (approximately 3% on average for both categories; note that the averages reported in the text of this section represent mean values, aggregating across both the immediate and delayed training groups).

After training, we observed that participants changed their behavior to more closely match the supportive psychotherapy guidelines taught. As evidenced by the between-group comparisons, the course had strong effects on several of these baseline behaviors in line with our hypotheses. Helpers decreased their overall volume of speech (*d=*−1.5 for within-person change from pretraining to posttraining) from an average of 163 (SD 82) utterances per 30 min of discussion time before training to an average of 34 (SD 36) utterances posttraining. They increased their frequency of restatements (*d*=1.0); on average, restatements formed 3% (SD 4%) of the session at baseline and grew to 25% (SD 22%) posttraining. Helpers also decreased average attempts to influence the talker (*d*=−1.6) from 34% (SD 18%) of the session at baseline to 8% (SD 14%) posttraining. Taking the course decreased self-disclosure (*d*=−0.8) from 18% (SD 14%) to 4% (SD 9%) and speech behaviors in the *other* category (*d*=−0.8) from 48% (SD 15%) to 34% (SD 19%).

Evidence of an effect on open-ended questions was more equivocal: open-ended questions increased (*d*=0.8) from around 3% (SD 3%) to 9% (SD 8%), but the 95% CI for the visit by condition interaction included an odds ratio of 1, so one cannot claim with certainty that change was because of the course. There was no strong indication that participants changed closed-ended questions (*d*=0.2) or expressions of sympathy (*d*=−0.1).

#### RQ 2: Does the Course Increase Helpers’ Overall Adherence to Helping Skills Guidelines?

Adherence scores greatly increased from an average of −20.0 (SD 7.0) at pretraining to +2.6 (SD 10.2) at posttraining (*d*=2.1). The between-group analysis showed that this change can be causally attributed to training.

#### RQ 3: Does the Talker’s Perception of Session Helpfulness Increase After Taking the Course?

On the CSRS task reactions scale, participants indicated that they perceived more progress in developing insight and solving problems in their sessions after taking the course (*d*=1.1), going from an average score of 5.4 (SD 1.5) to an average score of 7.0 (SD 1.6). In contrast, there was no reliable evidence for change in the CSRS relationship reactions subscale, which represents feelings of understanding and support between peers (*d*=0.3); the average score was 6.8 (SD 1.5) pretraining and 7.1 (SD 1.9) posttraining.

### Accounting for Attrition as a Potential Confound

Overall, 30% (9/30) of dyads withdrew, 4 of the 9 (44%) from the immediate training condition and 5 of the 9 (56%) from the delayed training condition (not a significant difference; χ^2^_1_=0.1; *P*=.79). The most endorsed reasons for attrition were difficulty finding time or motivation to work on the course, stress from the additional workload conferred by the course, and interference from unanticipated life events. There were no differences in psychological symptoms or stress between those who withdrew and those who did not, and there were no differences in demographic characteristics. Individuals who withdrew were more likely to report past psychotherapy (66.7% vs 16.7%; χ^2^_1_=12.3; *P*<.001) and current psychiatric medication use (33.3% vs 7.1%; χ^2^_1_=6.8; *P*=.02) than individuals who completed the study. There was also a marginally significant difference in household income (divided by the square root of the number of household members to adjust for household size and economies of scale), with those who withdrew coming from higher-income households (median US $61,500 for withdrawers and US $35,800 for completers; two-tailed t_25.4_=1.94; *P=*.06).

In most trials, participants decide whether to withdraw of their own accord, raising the possibility that differences in outcome are due to self-selection rather than to the effects of the intervention. In this study, attrition from the study took place pairwise: if one participant wished to exit the study, that person’s peer left as well; consequently, withdrawal from the study was not perfectly correlated with intention to remain in the study. All participants (regardless of whether they left the study prematurely) retrospectively rated on a 10-point Likert scale how much they had wanted to withdraw versus remain. Withdrawers indicated a greater desire to leave the study (mean 5.8, SD 2.1) than completers (mean 4.5, SD 2.1; *d*=0.60), although the difference failed to achieve statistical significance (*P*=.09). Thus, there is still some possibility of self-selection affecting the results, such that participants who withdrew from the study might have shown no skill improvement, attenuating effect sizes.

By statistically controlling for the desire to withdraw, one can potentially model the missing data mechanism so that the assumption of missingness at random is met, reducing or eliminating bias in effect estimates [96]. Therefore, we conducted a post-hoc analysis in which we re-estimated the models used to investigate changes from pre- to posttraining, now while controlling for desire to withdraw and the interaction between desire and time point. All of the 95% CIs for effects of motivation to withdraw included 0, and other coefficients remained similar, suggesting that attrition is unlikely to be a meaningful source of bias in the results. The detailed results are presented in Multimedia Appendix 1.

## Discussion

### Principal Findings

The goal of this study was to test the efficacy of a web-based course for teaching counseling skills to nonprofessionals, including those with elevated psychological symptoms. The course caused participants to change most of their helper speech behaviors in the hypothesized directions. Participants spoke less during a mock CMH session and they spent less time talking about themselves, suggesting that they learned to focus their attention on the talker. They increased their use of restatements and decreased their attempts to influence the talker. They also slightly increased their use of open-ended questions, although there was insufficient evidence that this increase was caused by taking the course, and there was no decrease in closed-ended questions. Overall, participants showed substantial increases in aggregate adherence scores. In addition, participants reported more progress in problem solving and insight during counseling sessions after taking the course, which may indicate that peer counseling using this model could improve mental health.

These findings provide cause for optimism that nonprofessionals can learn to deliver therapeutic ingredients via primarily self-directed web-based courses. This model—reciprocal peer delivery of techniques derived from supportive psychotherapy that are taught via a self-directed web-based course—has a variety of advantages that enable it to address gaps in traditional mental health care. First, it addresses practical barriers to treatment access because it does not require working with professionals (who often have limited availability), has no financial cost, and can be conducted in flexible times and places. Second, it addresses attitudinal barriers by not requiring participants to identify as mentally ill or see themselves as needing help (instead, they are in an egalitarian relationship). Third, it addresses gaps in the treatment of subclinical symptoms and distress by using a transdiagnostic treatment (supportive psychotherapy) that is appropriate even in the absence of a diagnosable mental illness. In addition to these gap-addressing features, it has the additional potential to increase feelings of intimacy and perceived social support and to provide the psychological benefits of delivering care in a way that is not present in psychotherapy with a professional.

The evidence suggests that supportive psychotherapy is efficacious for a variety of conditions, but it may not be as powerful as other treatments (eg, those that target specific symptoms of a disorder), especially if delivered by peers. However, even if such peer-delivered interventions are not as powerful as those delivered by professionals and even if only a subset of laypeople can learn the skills, disseminating therapeutic ingredients through nonprofessionals could improve public health by reaching large numbers of people who might not otherwise receive mental health support. One can imagine numerous permutations of peer-delivered interventions for the many settings where need is great and access or willingness to use traditional psychotherapy is low. Continued research and development of web-based training programs such as CMH that use the reciprocal peer counseling model is warranted.

Despite the clear impact of the course on most behaviors and perceived helpfulness, this study also suggests that refinements might be needed to improve its efficacy and reach. Perhaps the largest concern is attrition: 30% of the participants chose to withdraw from the study. Although this value is comparable with dropout rates for psychotherapy trials [97,98] and trials of smartphone apps to treat anxiety and depression [48] and is low relative to the 80% to 90% rate often cited for massive open online courses [99], it suggests the course could be modified to increase motivation or decrease learner burden. Interestingly, individuals who withdrew from the study were no more psychologically distressed or symptomatic than those who continued, but they were more likely to have experience using professional mental health services and had marginally higher household income. They may have had greater access to or comfort with traditional treatment and thus felt a less pressing need to learn an alternative tool for mental health support. Regardless of their reasons for withdrawing, some participants clearly found the course burdensome; although the course had no financial cost, the version used in this study requires considerable time and effort. Maximizing scalability for CMH and related courses means minimizing the time and effort cost without compromising efficacy. Anecdotally, participants seemed to find the lessons on talking more onerous than the lessons on helping; thus, for future iterations of the course, we plan to reduce or eliminate the talking skills lessons and replace them with real-time, in-session topic prompts [100], in addition to using participant feedback to make the helper lessons more enjoyable.

Finally, reported feelings of interpersonal closeness and support assessed via the CSRS relationship reactions subscale remained stable (there was a small increase, but a zero increase was a credible value in the Bayesian models). This may be attributable to a ceiling effect: participants’ ratings of their relationship-related perceptions in the first mock session were high. It is also likely that measurable changes in perceptions of support giving in relationships require more than one counseling session, especially in established relationships in which perceptions of the other person’s supportive behavior may draw on information from numerous interactions. Nevertheless, future iterations of the course can draw on close relationships and communication research to identify more ways to foster feelings of closeness and support.

### Generalizability

The study’s sample was fairly culturally diverse, with approximately 40% of participants identifying as non-White (compared with about 25% of the US population), and more than 1 in 5 participants born outside the United States. Several were recent English language learners, and one of these informally commented that she found the English of the course accessible and useful for practicing her English skills. The success of the course with this sample suggests that it is at least effective in teaching people with diverse cultural and linguistic backgrounds. However, this does not mean that it will be successful in improving the mental health of individuals from all cultures, especially considering that culturally adapted psychotherapy is more effective [101] and that there are cultural differences in preferred and delivered social support styles [102,103].

In addition, the course’s educational efficacy for individuals with less formal education or technology experience remains unknown because most participants were college educated or current students and, thus, may have been particularly well-equipped to learn from the course. Adaptations may be warranted for other populations. Fortunately, even if delivering these skills in this format to individuals with less education or comfort with technology is found to be impractical, CMH could still have a public health impact. It could, for example, be deployed with college students, addressing rising psychological distress and the shortage of mental health services on college campuses [104]. However, it would be ideal to make CMH accessible to as many individuals as possible; thus, further research must assess whether a redesign is needed to reach those without a college education.

Although we made efforts to make the study as ecologically valid as possible (eg, framing compensation as payment for study visits rather than completing the course; encouraging participants to speak “as [they] really would in everyday life, without trying to impress anybody”), it must be acknowledged that learners might engage with the course material differently or adhere less to the supportive psychotherapy skills if they are not monitored by research staff or financially incentivized to participate. This limitation to ecological validity highlights the need to make the course truly intrinsically motivating to facilitate adherence.

### Limitations and Future Directions

In addition to some limitations to generalizability, the study’s scope (ie, assessing the impact of the course on skill performance immediately after training) limits the conclusions that can be drawn. In particular, the mental health impact of applying CMH skills has not been rigorously investigated. As a proxy for the impact of the intervention, participants rated the perceived helpfulness of their sessions, but there is no guarantee that what participants viewed as helpful in the short run would have positive effects on psychological symptoms in the long run. Furthermore, because participants were not blinded to condition, the measured increase in perceived session helpfulness could be driven by a placebo effect or experimenter demand.

Furthermore, because the mental health effects of engaging in these peer counseling sessions were not assessed beyond the immediate postsession reaction, we could not thoroughly assess any harm or risks that could result from participating. For example, it is possible that peers could use these sessions as an opportunity to air grievances with each other or to gossip about mutual acquaintances, damaging their relationships. We attempted to mitigate this possibility by including instructions in the course that (1) discourage partnering with a peer with whom one has a contentious or familial relationship and (2) proscribe discussing a stressor that directly involves one’s partner (instead, we encouraged learners to find a neutral third party with whom to discuss such topics).

Peers could also disclose extremely troubling or traumatic material, or indications of risk such as suicidal thoughts, to which nonprofessionals rarely have the skills to respond. For the purposes of this study, participants were instructed not to discuss such material with their peers, but instead to contact a professional or the research staff if they needed to talk about such topics. (They were provided with extensive local referral information.) If the course were launched to the general public, additional safeguards would need to be put in place, such as easy access to crisis hotlines along with materials that have been empirically demonstrated to increase utilization of crisis services [105] and instructions for responding to a suicidal peer, among others.

A potentially more common risk is that peer counseling partners engage in co-rumination, a process that involves repetitive discussion and speculation about problems and has demonstrable negative as well as positive effects (ie, on relationships and mental health) [106-108]. This risk was one of the reasons we included instructions for the talker that encouraged talkers to identify proactive coping or problem-solving actions within a session or two rather than repeatedly rehashing problems. Despite the inclusion of these mitigations, it is impossible to perfectly control peer counseling behaviors, so harm could result from participating. The question is whether the benefits outweigh these risks and whether these harms would have occurred anyway in the absence of a formal peer counseling program (ie, friends may disclose distressing material or suicidal thoughts in everyday life, and the addition of a structured counseling program may not affect the frequency of such disclosures).

An additional limitation of this study is the absence of a follow-up timepoint to assess the durability of the training. It is possible that as they engage in repeated reciprocal peer counseling sessions over time, CMH users may forget the material or drift toward their typical interaction styles. Periodic self-assessments and booster training sessions may be needed to maintain skills over time.

Future work should aim to remedy the limitations of this investigation. As a first step, the course must be revised to reduce the time and effort required to complete it (eg, by replacing the talker lessons with in-session discussion prompts). Next, it is essential to assess the longer-term mental health effects as well as risks of harm that result from engaging in repeated CMH sessions with a peer. These studies should be conducted in naturalistic settings, to the degree possible, while maintaining some monitoring (eg, through regular assessment) for ethical reasons. These longer-term studies must also assess whether skills erode over time and whether such erosion can be prevented with self-assessment tools (to check whether one has followed guidelines) and/or booster training sessions.

To maximize its reach, CMH is also likely to require tailoring to specific populations (eg, individuals with less education or experience with technology, particular cultural groups, or others). The tailoring process can begin with qualitative research (eg, focus groups, interviews, and pilot testing) to drive initial revisions to the course, followed by experimental assessments of the updated course’s effects on skills and the longer-term mental health effects. Importantly, early steps in this process may reveal that CMH’s defining features do not adequately address the unique needs or barriers experienced by a particular group and that it is necessary to develop entirely different innovative interventions.

When the course is eventually launched at scale, frequent A/B testing (controlled experiments comparing two different versions of a website or software) can be used to fine-tune it to be as effective as possible, to tailor it further to specific populations, to eliminate exercises that do not increase skill, and to make other refinements.

### Conclusions

This study demonstrates the feasibility of teaching empirically supported counseling skills to pairs of nonprofessionals via a highly scalable web-based course. Although the model may not be able to reach all populations, this study demonstrates the potential of the CMH model to fill important gaps in the current mental health care system. Further research and refinement are necessary to assess the mental health effects of the course and to ensure that it is effective for diverse groups. Our results underscore that reciprocal, peer-delivered interventions disseminated via web-based courses have the potential to fill gaps in mental health care, thus enabling evidence-based treatment ingredients to reach individuals who might otherwise not be served by the existing mental health care system.

## Appendix

Multimedia Appendix 1 Supplementary data analytic methods and results.

Multimedia Appendix 2 Crowdsourcing Mental Health Session Reaction Scale.

## Abbreviations

BMT: behavior modeling training
BSI: Brief Symptom Inventory
CI: credibility interval
CMH: Crowdsourcing Mental Health
CSRS: Crowdsourcing Mental Health Session Reaction Scale
PSS: Perceived Stress Scale
RQ: research question
