# Supplementary material for: Nonprofessional Peer Support to Improve Mental Health: Randomized Trial of a Scalable Web-Based Peer Counseling Course
Source: J Med Internet Res. 2020 Sep 21;22(9):e17164. doi: 10.2196/17164 (PMC7536598; doi:10.2196/17164)
Supplement: Multimedia Appendix 1 [file jmir_v22i9e17164_app1.docx]

**Supplemental Methods**

**Skill Performance Coding System Methods and Psychometrics**

Prior to coding, video recordings of the sessions were transcribed and stripped of cues indicative of the time point or condition, allowing raters to remain blind to whether participants had taken the course prior to the session. Undergraduate research assistant coders spent at least 30 hours in training, including didactic workshops, practice, and discussion. Only research assistants showed good interrater reliability with the first author on several practice transcripts were permitted to code study transcripts. Two raters coded each transcript separately, then met to compare responses and resolve disagreements.

Interrater agreement was computed using Cohen’s (1960) kappa. For choosing among the six mutually exclusive categories, the average kappa *prior* to resolving disagreements was .82 (*SD* = .11), and the average kappa for deciding whether a unit included influencing was .63 (*SD* = .28). To estimate the reliability of the final codes (i.e., the codes after disagreements were resolved), kappa was calculated between the final codes and the first author’s own codes for a sample of eight sessions. The mean kappa for these eight sessions was .88 (*SD* = .07) for the six mutually exclusive categories and .79 (*SD* = .34) for influencing. Thus, reliability as assessed via the kappa statistic was acceptable. Importantly, the kappa statistic, which assesses whether raters agree on the categorization of each individual unit, sets a much higher bar for agreement than is necessary for this study. The outcome variables are based on the total number or proportion of sentence-units in each category, so it would not matter if raters disagreed on the classification of individual units, as long as they concluded that approximately the same number fell in each category. Given the average kappa values, the coding system appears to have been applied with an acceptable level of measurement error.

**CMH Session Reaction Scale (CSRS) Development**

The CMH Session Reaction Scale (CSRS) was modified from the Revised Session Reaction Scale (RSRS; Elliott, 1993), an instrument used for clients to rate psychotherapy sessions that covers the potential mechanisms of supportive peer counseling: awareness and insight; progress toward solving problems; immediate emotional relief; self-acceptance; and feeling understood by, supported by, and closer to the helper. To create the CSRS, four RSRS items that were less relevant to CMH were deleted, wording was simplified, and the term “therapist” was replaced with “partner” (which was the word used with participants to refer to their selected peers).

Items were expected to load on two subscales: task reactions (progress towards resolution of the problem through insight, emotional relief, or problem-solving) and relationship reactions (feeling understood by, connected to, and supported by one’s partner). The instrument was subjected to confirmatory factor analysis using the R package *lavaan* (Rosseel, 2012). Responses to negatively-worded (i.e., reverse-scored) items were extremely skewed, such that endorsement of negative responses to a session was rare; these items loaded poorly on their scales, so they were dropped. One additional item that loaded poorly on the task reactions scale was also removed. The remaining six task reactions items and three relationship reactions items all had standardized loadings of at least .5 on their respective scales. The final model was an adequate fit to the data (*χ*^2^[26] = 36.95, *p* = .075, RMSEA = .057, GFI = .996, SRMR = .042, CFI= .998). Scales were scored by computing the mean of the items, resulting in a theoretical range of 1 to 9 for both. Internal consistency of each scale was good at all laboratory visits (coefficient α .86-.92 for task reactions and .84-.95 for relationship reactions).

**Data Analytic Details**

**Between-subjects analysis.** To investigate whether the magnitude of change between the first two visits was greater in the immediate condition than in the delayed condition, we ran a three-level model with the following form, predicting the value of the outcome variable at visit *i* for person *j* in dyad *k*.

Level 1 (within-person):

Outcome*_ijk_* = *β*_0_*_jk_* + *β*_1_*_jk_*(Visit)*_ijk_* (1)

Level 2 (between-person, within-dyad):

*β*_0_*_jk_* = *γ*_00_*_k_* + *u*_0_*_jk_* (2)

*β*_1_*_jk_* = *γ*_10_*_k_* + *u*_1_*_jk_* (3)

Level 3 (between-dyad):

*γ*_00_*_k_* = *δ*_000_ + *δ*_001_(Condition)*_k_* + *v*_0_*_k_* (4)

*γ*_10_*_k_* = *δ*_100_ + *δ*_101_(Condition)*_k_* + *v*_1_*_k_* (5)

The combined equation, thus, is

Outcome*_ijk_* = *δ*_000_ + *δ*_001_(Condition)*_k_* + *δ*_100_(Visit)*_ijk_* + *δ*_101_(Condition)*_k_*(Visit)*_ijk_* +

*v*_0_*_k_* + *u*_0_*_jk_* + *v*_1_*_k_*(Visit)*_ijk_* + *u*_1_*_jk_*(Visit)*_ijk_* (6)

Condition was coded with delayed as 0 and immediate as 1, and visit was coded with the first visit as -1 and the second visit as 0. Thus, the intercept represents the value of the outcome variable for the delayed condition at the second visit; the effect of condition represents the difference between the immediate and delayed conditions at the second visit; the effect of visit represents the change from visit 1 to visit 2 in the delayed group; and the interaction between visit and condition represents the magnitude by which change from visit 1 to visit 2 differed between the two conditions. If there is an effect of the course, but no effect of waiting, one would expect a near-zero coefficient for the effect of visit and positive coefficients for condition and visit-condition interaction if the course increases a variable (or negative coefficients if the course decreases the variable). Results for these models appear in Table S1.

**Within-subjects analysis.** We used a simpler version of the above model to estimate the magnitude of change from pre-training to post-training aggregating across both conditions.

Level 1 (within-person):

Outcome*_ijk_* = *β*_0_*_jk_* + *β*_1_*_jk_*(Training)*_ijk_* (7)

Level 2 (between-person, within-dyad):

*β*_0_*_jk_* = *γ*_00_*_k_* + *u*_0_*_jk_* (8)

*β*_1_*_jk_* = *γ*_10_*_k_* + *u*_1_*_jk_* (9)

Level 3 (between-dyad):

*γ*_00_*_k_* = *δ*_000_ + *v*_0_*_k_* (10)

*γ*_10_*_k_* = *δ*_100_ + *v*_1_*_k_* (11)

Combined:

Outcome*_ijk_* = *δ*_000_ + *v*_0_*_k_* + *u*_0_*_jk_* + *δ*_100_(Training)*_ijk_* + *v*_1_*_k_*(Training)*_ijk_* +

*u*_1_*_jk_*(Training)*_ijk_* (12)

Training was coded as -1 for pre-training and 0 for post-training, so that the intercept can be interpreted as the post-training value of the outcome variable and the effect of training as the amount of change from pre- to post-training. Results of these models appear in Table S2.

**Analysis controlling for desire to withdraw from the study.** Finally, we ran a series of models to determine whether desire to withdraw from the study was associated with one’s performance at the post-training visit or with the magnitude of change from pre- to post-training. To do so, we added the effect of desire to withdraw and the interaction of desire and training to the above model. Desire to withdraw was rated on a 1-10 scale. Results of these models appear in Table S3.

All models were run using the R package *brms* (Bürkner, 2017)*,* which implements Bayesian multilevel models in the programming language Stan (Stan Development Team, 2016). For each type of outcome, the models differed in the distribution of the outcome variable, the link function, and the scale of the prior distributions. The proportions of sentence-units falling in each category were modeled with a binomial distribution and a logistic link function. The probability of passing was modeled with a Bernoulli distribution and a logistic link function. The number of sentence-units and the number of competence criteria met were modeled with a Poisson distribution and a log link function. Finally, the competence score and CSRS scores were modeled with a Gaussian distribution and an identity link function. For all models, priors were chosen to ascribe fairly equal probability to all values within reason given the scale of the data. Consequently, the posterior distributions were influenced almost exclusively by the data.

The Stan language samples the posterior distribution using Hamiltonian Monte Carlo, which converges faster than alternative algorithms (Neal, 2011). For each model, we ran four chains, each with 1,000 steps of warm-up and thinning of 2, saving a total of 4,000 sampled values per model. There was evidence that all models achieved adequate convergence and resembled the target distributions (all *R̂* values < 1.1; Gelman & Rubin, 1992).

| **Supplemental Results**  Table S1 | | | | | | | | | | | |
| --- | --- | --- | --- | --- | --- | --- | --- | --- | --- | --- | --- |
| *Bayesian Multilevel Models Testing Differences Between Immediate and Delayed Conditions at First Two Laboratory Visits* | | | | | | | | | | | |
|  |  |  |  |  |  |  |  |  |  |  |  |
|  | Total units | | |  | Restatement | | |  | Influencing | | |
| Parameter | *M* est | *SD* est | 95% CI |  | *M* est | *SD* est | 95% CI |  | *M* est | *SD* est | 95% CI |
| Fixed |  |  |  |  |  |  |  |  |  |  |  |
| Intercept | 5.11 | 0.17 | 4.76, 5.42 |  | -4.52 | 0.38 | -5.29, -3.80 |  | -0.52 | 0.19 | -0.89, -0.15 |
| Visit | 0.07 | 0.17 | -0.26, 0.40 |  | -0.62 | 0.48 | -1.60, 0.33 |  | -0.02 | 0.20 | -0.41, 0.38 |
| Condition | -2.15 | 0.26 | -2.67, -1.63 |  | 3.06 | 0.57 | 1.96, 4.20 |  | -2.46 | 0.34 | -3.16, -1.83 |
| Visit X condition | -1.97 | 0.25 | -2.47, -1.46 |  | 3.35 | 0.70 | 1.93, 4.78 |  | -2.00 | 0.36 | -2.71, -1.29 |
| Random |  |  |  |  |  |  |  |  |  |  |  |
| Person intercept SD | 0.51 | 0.09 | 0.37, 0.72 |  | 0.84 | 0.25 | 0.42, 1.42 |  | 0.75 | 0.13 | 0.52, 1.03 |
| Person visit SD | 0.49 | 0.09 | 0.34, 0.69 |  | 1.32 | 0.30 | 0.80, 1.97 |  | 0.88 | 0.14 | 0.64, 1.19 |
| Person intercept-visit correlation | 0.43 | 0.18 | 0.05, 0.74 |  | 0.54 | 0.21 | 0.05, 0.86 |  | 0.37 | 0.18 | -0.01, 0.67 |
| Dyad intercept SD | 0.51 | 0.15 | 0.18, 0.81 |  | 1.09 | 0.32 | 0.39, 1.72 |  | 0.37 | 0.20 | 0.02, 0.77 |
| Dyad visit SD | 0.49 | 0.15 | 0.15, 0.78 |  | 1.24 | 0.45 | 0.24, 2.07 |  | 0.35 | 0.22 | 0.02, 0.81 |
| Dyad intercept-visit correlation | 0.82 | 0.21 | 0.25, 0.99 |  | 0.69 | 0.30 | -0.25, 0.98 |  | 0.01 | 0.54 | -0.94, 0.92 |
|  |  |  |  |  |  |  |  |  |  |  |  |
|  | Open-ended questions | | |  | Closed-ended questions | | |  | Self-disclosure | | |
| Parameter | *M* est | *SD* est | 95% CI |  | *M* est | *SD* est | 95% CI |  | *M* est | *SD* est | 95% CI |
| Fixed |  |  |  |  |  |  |  |  |  |  |  |
| Intercept | -3.79 | 0.26 | -4.32, -3.32 |  | -2.26 | 0.15 | -2.57, -1.97 |  | -1.85 | 0.36 | -2.53, -1.14 |
| Visit | 0.47 | 0.28 | -0.10, 1.03 |  | 0.27 | 0.17 | -0.07, 0.60 |  | -0.07 | 0.30 | -0.66, 0.52 |
| Condition | 0.81 | 0.41 | 0.02, 1.61 |  | 0.25 | 0.25 | -0.23, 0.73 |  | -2.64 | 0.66 | -4.05, -1.47 |
| Visit X condition | 0.34 | 0.43 | -0.52, 1.17 |  | -0.46 | 0.27 | -1.01, 0.07 |  | -2.41 | 0.58 | -3.68, -1.37 |
| Random |  |  |  |  |  |  |  |  |  |  |  |
| Person intercept SD | 0.96 | 0.19 | 0.62, 1.38 |  | 0.66 | 0.10 | 0.49, 0.88 |  | 1.36 | 0.29 | 0.86, 1.99 |
| Person visit SD | 0.97 | 0.22 | 0.57, 1.44 |  | 0.74 | 0.11 | 0.55, 0.98 |  | 0.96 | 0.23 | 0.57, 1.45 |
| Person intercept-visit correlation | 0.71 | 0.15 | 0.32, 0.92 |  | 0.44 | 0.15 | 0.10, 0.69 |  | 0.61 | 0.19 | 0.17, 0.87 |
| Dyad intercept SD | 0.44 | 0.24 | 0.03, 0.93 |  | 0.19 | 0.13 | 0.01, 0.49 |  | 0.83 | 0.42 | 0.07, 1.68 |
| Dyad visit SD | 0.35 | 0.23 | 0.02, 0.86 |  | 0.13 | 0.10 | 0.01, 0.38 |  | 0.70 | 0.35 | 0.07, 1.42 |
| Dyad intercept-visit correlation | 0.12 | 0.56 | -0.92, 0.95 |  | 0.20 | 0.58 | -0.93, 0.98 |  | 0.60 | 0.43 | -0.67, 0.99 |

| Table S1 Continued |  |  |  |  |  |  |  |  |  |  |  |
| --- | --- | --- | --- | --- | --- | --- | --- | --- | --- | --- | --- |
|  |  |  |  |  |  |  |  |  |  |  |  |
|  | Sympathy | | |  | Other | | |  | Pass/fail | | |
| Parameter | *M* est | *SD* est | 95% CI |  | *M* est | *SD* est | 95% CI |  | *M* est | *SD* est | 95% CI |
| Fixed |  |  |  |  |  |  |  |  |  |  |  |
| Intercept | -2.35 | 0.28 | -2.9, -1.78 |  | 0.08 | 0.20 | -0.34, 0.45 |  | -19.03 | 7.36 | -36.46, -7.53 |
| Visit | -0.12 | 0.19 | -0.50, 0.26 |  | -0.09 | 0.18 | -0.45, 0.27 |  | 13.03 | 7.66 | -1.32, 28.33 |
| Condition | 0.42 | 0.43 | -0.41, 1.26 |  | -0.80 | 0.31 | -1.42, -0.19 |  | 15.79 | 6.25 | 4.69, 29.12 |
| Visit X condition | 0.08 | 0.31 | -0.53, 0.67 |  | -0.44 | 0.29 | -1.02, 0.12 |  | 10.23 | 7.83 | -5.26, 25.63 |
| Random |  |  |  |  |  |  |  |  |  |  |  |
| Person intercept SD | 0.63 | 0.14 | 0.40, 0.98 |  | 0.52 | 0.10 | 0.36, 0.74 |  | 2.30 | 2.08 | 0.07, 7.67 |
| Person visit SD | 0.55 | 0.13 | 0.32, 0.83 |  | 0.36 | 0.11 | 0.18, 0.62 |  | 4.25 | 3.38 | 0.17, 12.66 |
| Person intercept-visit correlation | 0.17 | 0.28 | -0.43, 0.65 |  | 0.26 | 0.28 | -0.34, 0.70 |  | 0.08 | 0.58 | -0.94, 0.96 |
| Dyad intercept SD | 0.94 | 0.23 | 0.55, 1.43 |  | 0.62 | 0.16 | 0.32, 0.94 |  | 8.63 | 5.23 | 2.15, 21.71 |
| Dyad visit SD | 0.46 | 0.20 | 0.06, 0.86 |  | 0.58 | 0.15 | 0.31, 0.89 |  | 5.80 | 5.01 | 0.18, 18.56 |
| Dyad intercept-visit correlation | 0.64 | 0.32 | -0.33, 0.99 |  | 0.79 | 0.18 | 0.41, 0.99 |  | 0.28 | 0.56 | -0.89, 0.98 |
|  |  |  |  |  |  |  |  |  |  |  |  |
|  | Competence score | | |  | CSRS task reactions | | |  | CSRS relationship reactions | | |
| Parameter | *M* est | *SD* est | 95% CI |  | *M* est | *SD* est | 95% CI |  | *M* est | *SD* est | 95% CI |
| Fixed |  |  |  |  |  |  |  |  |  |  |  |
| Intercept | -0.55 | 0.14 | -0.82, -0.28 |  | -0.22 | 0.19 | -0.61, 0.15 |  | -0.17 | 0.24 | -0.64, 0.30 |
| Visit | -0.10 | 0.15 | -0.39, 0.19 |  | -0.01 | 0.20 | -0.40, 0.37 |  | -0.07 | 0.19 | -0.46, 0.31 |
| Condition | 2.18 | 0.20 | 1.78, 2.58 |  | 1.02 | 0.29 | 0.47, 1.59 |  | 0.41 | 0.35 | -0.28, 1.13 |
| Visit X condition | 1.90 | 0.21 | 1.48, 2.32 |  | 1.00 | 0.29 | 0.41, 1.56 |  | 0.20 | 0.29 | -0.38, 0.77 |
| Random |  |  |  |  |  |  |  |  |  |  |  |
| Person intercept SD | 0.22 | 0.14 | 0.01, 0.51 |  | 0.71 | 0.15 | 0.43, 1.02 |  | 0.79 | 0.16 | 0.50, 1.12 |
| Person visit SD | 0.29 | 0.18 | 0.01, 0.68 |  | 0.34 | 0.26 | 0.01, 0.91 |  | 0.34 | 0.23 | 0.01, 0.81 |
| Person intercept-visit correlation | 0.12 | 0.52 | -0.91, 0.91 |  | 0.22 | 0.47 | -0.85, 0.93 |  | 0.34 | 0.42 | -0.74, 0.95 |
| Dyad intercept SD | 0.37 | 0.12 | 0.12, 0.61 |  | 0.22 | 0.16 | 0.01, 0.57 |  | 0.60 | 0.24 | 0.08, 1.04 |
| Dyad visit SD | 0.22 | 0.14 | 0.01, 0.53 |  | 0.29 | 0.19 | 0.02, 0.69 |  | 0.46 | 0.20 | 0.05, 0.85 |
| Dyad intercept-visit correlation | 0.38 | 0.48 | -0.80, 0.97 |  | 0.23 | 0.56 | -0.91, 0.97 |  | 0.48 | 0.42 | -0.72, 0.96 |
| Residual SD | 0.40 | 0.11 | 0.12, 0.55 |  | 0.58 | 0.14 | 0.21, 0.79 |  | 0.42 | 0.14 | 0.13, 0.65 |
| *Note*. 95% CI = 95% credibility interval; CSRS = CMH Session Reaction Scale; *M* est = mean of posterior distribution; *SD* = standard deviation of posterior distribution | | | | | | | | | | | |

| Table S2 | | | | | | | | | | | |
| --- | --- | --- | --- | --- | --- | --- | --- | --- | --- | --- | --- |
| *Bayesian Multilevel Models Estimating Pre- to Post-Training Change* | | | | | | | | | | | |
|  |  |  |  |  |  |  |  |  |  |  |  |
|  | Total units | | |  | Restatement | | |  | Influencing | | |
| Parameter | *M* est | *SD* est | 95% CI |  | *M* est | *SD* est | 95% CI |  | *M* est | *SD* est | 95% CI |
| Fixed |  |  |  |  |  |  |  |  |  |  |  |
| Intercept | 3.1 | 0.17 | 2.77, 3.43 |  | -1.53 | 0.27 | -2.07, -1.00 |  | -3.21 | 0.38 | -4.08, -2.54 |
| Training | -1.88 | 0.17 | -2.21, -1.54 |  | 2.8 | 0.37 | 2.07, 3.55 |  | -2.46 | 0.37 | -3.29, -1.81 |
| Random |  |  |  |  |  |  |  |  |  |  |  |
| Person intercept SD | 0.82 | 0.14 | 0.58, 1.11 |  | 1.02 | 0.22 | 0.65, 1.51 |  | 1.60 | 0.36 | 1.00, 2.41 |
| Person training SD | 0.78 | 0.15 | 0.52, 1.09 |  | 1.75 | 0.33 | 1.16, 2.44 |  | 1.46 | 0.34 | 0.89, 2.22 |
| Person intercept-training correlation | 0.83 | 0.07 | 0.66, 0.93 |  | 0.76 | 0.13 | 0.45, 0.94 |  | 0.85 | 0.08 | 0.66, 0.95 |
| Dyad intercept SD | 0.43 | 0.25 | 0.02, 0.93 |  | 0.85 | 0.33 | 0.18, 1.52 |  | 0.51 | 0.35 | 0.03, 1.36 |
| Dyad training SD | 0.47 | 0.26 | 0.03, 0.97 |  | 0.96 | 0.53 | 0.06, 2.05 |  | 0.48 | 0.34 | 0.03, 1.31 |
| Dyad intercept-training correlation | 0.67 | 0.46 | -0.79, 1.00 |  | 0.59 | 0.43 | -0.69, 0.99 |  | 0.34 | 0.56 | -0.87, 0.98 |
|  |  |  |  |  |  |  |  |  |  |  |  |
|  | Open-ended questions | | |  | Closed-ended questions | | |  | Self-disclosure | | |
| Parameter | *M* est | *SD* est | 95% CI |  | *M* est | *SD* est | 95% CI |  | *M* est | *SD* est | 95% CI |
| Fixed |  |  |  |  |  |  |  |  |  |  |  |
| Intercept | -2.52 | 0.18 | -2.89, -2.18 |  | -1.92 | 0.17 | -2.25, -1.61 |  | -4.93 | 0.71 | -6.58, -3.75 |
| Training | 1.29 | 0.20 | 0.90, 1.69 |  | 0.14 | 0.16 | -0.18, 0.44 |  | -3.02 | 0.71 | -4.64, -1.84 |
| Random |  |  |  |  |  |  |  |  |  |  |  |
| Person intercept SD | 0.65 | 0.16 | 0.35, 0.98 |  | 0.77 | 0.14 | 0.51, 1.07 |  | 2.42 | 0.66 | 1.37, 3.94 |
| Person training SD | 0.74 | 0.21 | 0.33, 1.16 |  | 0.70 | 0.15 | 0.43, 1.01 |  | 2.20 | 0.63 | 1.15, 3.62 |
| Person intercept-training correlation | 0.22 | 0.34 | -0.58, 0.73 |  | 0.51 | 0.19 | 0.07, 0.78 |  | 0.87 | 0.09 | 0.63, 0.97 |
| Dyad intercept SD | 0.38 | 0.22 | 0.03, 0.84 |  | 0.27 | 0.19 | 0.01, 0.71 |  | 0.75 | 0.56 | 0.03, 2.11 |
| Dyad training SD | 0.37 | 0.24 | 0.02, 0.88 |  | 0.21 | 0.16 | 0.01, 0.58 |  | 0.93 | 0.58 | 0.06, 2.33 |
| Dyad intercept-training correlation | 0.09 | 0.55 | -0.93, 0.94 |  | 0.32 | 0.57 | -0.89, 0.99 |  | 0.40 | 0.55 | -0.86, 0.99 |
|  |  |  |  |  |  |  |  |  |  |  |  |

| Table S2 Continued |  |  |  |  |  |  |  |  |  |  |  |
| --- | --- | --- | --- | --- | --- | --- | --- | --- | --- | --- | --- |
|  |  |  |  |  |  |  |  |  |  |  |  |
|  | Sympathy | | |  | Other | | |  | Pass/fail | | |
| Parameter | *M* est | *SD* est | 95% CI |  | *M* est | *SD* est | 95% CI |  | *M* est | *SD* est | 95% CI |
| Fixed |  |  |  |  |  |  |  |  |  |  |  |
| Intercept | -2.26 | 0.23 | -2.71, -1.81 |  | -0.74 | 0.17 | -1.1, -0.41 |  | -2.90 | 3.56 | -12.51, 1.75 |
| Training | -0.15 | 0.19 | -0.53, 0.21 |  | -0.68 | 0.17 | -1.02, -0.34 |  | 19.40 | 6.38 | 8.10, 33.18 |
| Random |  |  |  |  |  |  |  |  |  |  |  |
| Person intercept SD | 0.57 | 0.22 | 0.12, 1.02 |  | 0.53 | 0.16 | 0.26, 0.88 |  | 3.85 | 2.98 | 0.21, 11.22 |
| Person training SD | 0.65 | 0.18 | 0.29, 1.01 |  | 0.63 | 0.16 | 0.34, 0.96 |  | 3.88 | 3.15 | 0.15, 11.49 |
| Person intercept-training correlation | 0.38 | 0.35 | -0.52, 0.83 |  | 0.54 | 0.27 | -0.17, 0.87 |  | 0.21 | 0.58 | -0.91, 0.98 |
| Dyad intercept SD | 0.92 | 0.23 | 0.49, 1.42 |  | 0.58 | 0.19 | 0.16, 0.97 |  | 8.49 | 4.61 | 2.43, 20.14 |
| Dyad training SD | 0.36 | 0.23 | 0.02, 0.86 |  | 0.50 | 0.23 | 0.05, 0.93 |  | 5.83 | 4.83 | 0.20, 17.88 |
| Dyad intercept-training correlation | 0.31 | 0.48 | -0.81, 0.96 |  | 0.58 | 0.37 | -0.57, 0.96 |  | 0.41 | 0.54 | -0.85, 0.99 |
|  |  |  |  |  |  |  |  |  |  |  |  |
|  | Competence score | | |  | CSRS task reactions | | |  | CSRS relationship reactions | | |
| Parameter | *M* est | *SD* est | 95% CI |  | *M* est | *SD* est | 95% CI |  | *M* est | *SD* est | 95% CI |
| Fixed |  |  |  |  |  |  |  |  |  |  |  |
| Intercept | 0.98 | 0.12 | 0.74, 1.23 |  | 0.57 | 0.14 | 0.31, 0.85 |  | 0.18 | 0.18 | -0.18, 0.55 |
| Training | 1.64 | 0.13 | 1.39, 1.90 |  | 0.96 | 0.14 | 0.69, 1.26 |  | 0.28 | 0.17 | -0.06, 0.62 |
| Random |  |  |  |  |  |  |  |  |  |  |  |
| Person intercept SD | 0.46 | 0.20 | 0.05, 0.80 |  | 0.71 | 0.14 | 0.42, 1.01 |  | 0.95 | 0.16 | 0.62, 1.27 |
| Person training SD | 0.43 | 0.24 | 0.02, 0.83 |  | 0.41 | 0.28 | 0.02, 0.90 |  | 0.50 | 0.21 | 0.06, 0.87 |
| Person intercept-training correlation | 0.68 | 0.39 | -0.64, 0.99 |  | 0.28 | 0.43 | -0.83, 0.92 |  | 0.70 | 0.24 | 0.05, 0.98 |
| Dyad intercept SD | 0.31 | 0.17 | 0.03, 0.65 |  | 0.23 | 0.16 | 0.01, 0.53 |  | 0.40 | 0.24 | 0.02, 0.91 |
| Dyad training SD | 0.26 | 0.17 | 0.01, 0.62 |  | 0.24 | 0.16 | 0.01, 0.56 |  | 0.57 | 0.19 | 0.15, 0.95 |
| Dyad intercept-training correlation | 0.50 | 0.51 | -0.82, 0.99 |  | 0.34 | 0.55 | -0.88, 0.98 |  | 0.26 | 0.50 | -0.87, 0.93 |
| Residual SD | 0.39 | 0.12 | 0.11, 0.57 |  | 0.48 | 0.20 | 0.12, 0.76 |  | 0.34 | 0.14 | 0.06, 0.62 |
| *Note*. 95% CI = 95% credibility interval; CSRS = CMH Session Reaction Scale; *M* est = mean of posterior distribution; *SD* = standard deviation of posterior distribution | | | | | | | | | | | |

| Table S3 | | | | | | | | | | | |
| --- | --- | --- | --- | --- | --- | --- | --- | --- | --- | --- | --- |
| *Bayesian Multilevel Models Accounting for Desire to Withdraw from the Study* | | | | | | | | | | | |
|  |  |  |  |  |  |  |  |  |  |  |  |
|  | Total units | | |  | Restatement | | |  | Influencing | | |
| Parameter | *M* est | *SD* est | 95% CI |  | *M* est | *SD* est | 95% CI |  | *M* est | *SD* est | 95% CI |
| Fixed |  |  |  |  |  |  |  |  |  |  |  |
| Intercept | 2.90 | 0.37 | 2.16, 3.62 |  | -1.28 | 0.57 | -2.41, -0.19 |  | -4.10 | 0.82 | -5.86, -2.67 |
| Training | -2.04 | 0.37 | -2.78, -1.29 |  | 2.81 | 0.83 | 1.18, 4.41 |  | -3.31 | 0.78 | -4.98, -1.86 |
| Withdrawal motivation | 0.05 | 0.07 | -0.09, 0.19 |  | -0.06 | 0.11 | -0.28, 0.16 |  | 0.19 | 0.15 | -0.09, 0.51 |
| Training X withdrawal motivation | 0.03 | 0.07 | -0.11, 0.18 |  | 0.00 | 0.16 | -0.30, 0.31 |  | 0.18 | 0.15 | -0.10, 0.48 |
| Random |  |  |  |  |  |  |  |  |  |  |  |
| Person intercept SD | 0.82 | 0.15 | 0.57, 1.13 |  | 1.06 | 0.23 | 0.65, 1.55 |  | 1.61 | 0.34 | 1.02, 2.36 |
| Person training SD | 0.78 | 0.15 | 0.52, 1.10 |  | 1.79 | 0.34 | 1.16, 2.47 |  | 1.47 | 0.32 | 0.94, 2.19 |
| Person intercept-training correlation | 0.85 | 0.07 | 0.69, 0.94 |  | 0.76 | 0.12 | 0.47, 0.93 |  | 0.86 | 0.07 | 0.68, 0.95 |
| Dyad intercept SD | 0.46 | 0.26 | 0.03, 0.97 |  | 0.80 | 0.37 | 0.09, 1.54 |  | 0.45 | 0.33 | 0.02, 1.24 |
| Dyad training SD | 0.51 | 0.26 | 0.06, 1.02 |  | 0.98 | 0.55 | 0.06, 2.13 |  | 0.44 | 0.32 | 0.02, 1.21 |
| Dyad intercept-training correlation | 0.70 | 0.43 | -0.65, 0.99 |  | 0.58 | 0.45 | -0.72, 0.99 |  | 0.31 | 0.56 | -0.88, 0.98 |
|  |  |  |  |  |  |  |  |  |  |  |  |
|  | Open-ended questions | | |  | Closed-ended questions | | |  | Self-disclosure | | |
| Parameter | *M* est | *SD* est | 95% CI |  | *M* est | *SD* est | 95% CI |  | *M* est | *SD* est | 95% CI |
| Fixed |  |  |  |  |  |  |  |  |  |  |  |
| Intercept | -2.21 | 0.39 | -2.99, -1.44 |  | -1.65 | 0.38 | -2.42, -0.91 |  | -5.93 | 1.46 | -9.15, -3.36 |
| Training | 1.75 | 0.44 | 0.84, 2.60 |  | 0.24 | 0.37 | -0.5, 0.96 |  | -3.45 | 1.44 | -6.53, -0.87 |
| Withdrawal motivation | -0.07 | 0.08 | -0.23, 0.08 |  | -0.06 | 0.07 | -0.20, 0.09 |  | 0.20 | 0.26 | -0.33, 0.73 |
| Training X withdrawal motivation | -0.10 | 0.08 | -0.26, 0.08 |  | -0.02 | 0.07 | -0.16, 0.12 |  | 0.08 | 0.26 | -0.46, 0.61 |
| Random |  |  |  |  |  |  |  |  |  |  |  |
| Person intercept SD | 0.67 | 0.17 | 0.37, 1.02 |  | 0.79 | 0.15 | 0.53, 1.09 |  | 2.49 | 0.69 | 1.43, 4.05 |
| Person training SD | 0.71 | 0.22 | 0.28, 1.14 |  | 0.72 | 0.15 | 0.43, 1.03 |  | 2.26 | 0.66 | 1.21, 3.77 |
| Person intercept-training correlation | 0.16 | 0.35 | -0.65, 0.72 |  | 0.53 | 0.18 | 0.08, 0.79 |  | 0.89 | 0.08 | 0.69, 0.98 |
| Dyad intercept SD | 0.38 | 0.22 | 0.02, 0.86 |  | 0.26 | 0.18 | 0.01, 0.69 |  | 0.70 | 0.55 | 0.03, 2.09 |
| Dyad training SD | 0.37 | 0.24 | 0.02, 0.88 |  | 0.21 | 0.16 | 0.01, 0.60 |  | 0.93 | 0.58 | 0.06, 2.34 |
| Dyad intercept-training correlation | 0.06 | 0.55 | -0.94, 0.94 |  | 0.35 | 0.55 | -0.88, 0.99 |  | 0.41 | 0.54 | -0.85, 0.99 |
|  |  |  |  |  |  |  |  |  |  |  |  |

| Table S3 Continued |  |  |  |  |  |  |  |  |  |  |  |
| --- | --- | --- | --- | --- | --- | --- | --- | --- | --- | --- | --- |
|  |  |  |  |  |  |  |  |  |  |  |  |
|  | Sympathy | | |  | Other | | |  | Pass/fail | | |
| Parameter | *M* est | *SD* est | 95% CI |  | *M* est | *SD* est | 95% CI |  | *M* est | *SD* est | 95% CI |
| Fixed |  |  |  |  |  |  |  |  |  |  |  |
| Intercept | -2.53 | 0.45 | -3.40, -1.66 |  | -0.70 | 0.35 | -1.37, 0.03 |  | 0.70 | 8.40 | -17.93, 16.76 |
| Training | -0.83 | 0.39 | -1.62, -0.09 |  | -0.60 | 0.37 | -1.32, 0.14 |  | 9.83 | 8.43 | -6.42, 26.77 |
| Withdrawal motivation | 0.07 | 0.08 | -0.10, 0.22 |  | -0.01 | 0.07 | -0.15, 0.12 |  | -1.14 | 1.64 | -4.75, 1.82 |
| Training X withdrawal motivation | 0.15 | 0.08 | -0.01, 0.29 |  | -0.02 | 0.07 | -0.16, 0.13 |  | 14.73 | 6.89 | 2.76, 29.48 |
| Random |  |  |  |  |  |  |  |  |  |  |  |
| Person intercept SD | 0.52 | 0.23 | 0.08, 0.99 |  | 0.53 | 0.16 | 0.27, 0.88 |  | 8.08 | 5.22 | 1.01, 20.81 |
| Person training SD | 0.58 | 0.19 | 0.19, 0.95 |  | 0.62 | 0.16 | 0.33, 0.97 |  | 6.81 | 5.95 | 0.23, 21.98 |
| Person intercept-training correlation | 0.26 | 0.41 | -0.74, 0.81 |  | 0.53 | 0.26 | -0.12, 0.87 |  | 0.12 | 0.57 | -0.93, 0.97 |
| Dyad intercept SD | 0.97 | 0.24 | 0.53, 1.49 |  | 0.61 | 0.20 | 0.18, 1.01 |  | 13.99 | 7.72 | 3.10, 32.52 |
| Dyad training SD | 0.41 | 0.24 | 0.02, 0.91 |  | 0.54 | 0.23 | 0.07, 1.00 |  | 7.92 | 6.88 | 0.28, 26.24 |
| Dyad intercept-training correlation | 0.38 | 0.44 | -0.75, 0.96 |  | 0.62 | 0.35 | -0.45, 0.97 |  | 0.20 | 0.57 | -0.91, 0.98 |
|  |  |  |  |  |  |  |  |  |  |  |  |
|  | Competence score | | |  | CSRS task reactions | | |  | CSRS relationship reactions | | |
| Parameter | *M* est | *SD* est | 95% CI |  | *M* est | *SD* est | 95% CI |  | *M* est | *SD* est | 95% CI |
| Fixed |  |  |  |  |  |  |  |  |  |  |  |
| Intercept | 1.21 | 0.27 | 0.69, 1.74 |  | 1.17 | 0.34 | 0.50, 1.84 |  | 0.79 | 0.41 | -0.01, 1.59 |
| Training | 1.9 | 0.29 | 1.35, 2.46 |  | 1.28 | 0.34 | 0.61, 1.95 |  | 0.60 | 0.36 | -0.13, 1.29 |
| Withdrawal motivation | -0.06 | 0.05 | -0.16, 0.05 |  | -0.13 | 0.07 | -0.26, 0.00 |  | -0.14 | 0.08 | -0.30, 0.02 |
| Training X withdrawal motivation | -0.06 | 0.06 | -0.17, 0.06 |  | -0.07 | 0.07 | -0.20, 0.06 |  | -0.07 | 0.07 | -0.21, 0.07 |
| Random |  |  |  |  |  |  |  |  |  |  |  |
| Person intercept SD | 0.54 | 0.19 | 0.07, 0.84 |  | 0.68 | 0.15 | 0.39, 0.99 |  | 0.95 | 0.16 | 0.63, 1.25 |
| Person training SD | 0.52 | 0.23 | 0.04, 0.89 |  | 0.33 | 0.25 | 0.01, 0.87 |  | 0.50 | 0.21 | 0.07, 0.89 |
| Person intercept-training correlation | 0.77 | 0.28 | -0.23, 0.99 |  | 0.19 | 0.47 | -0.87, 0.91 |  | 0.67 | 0.26 | -0.04, 0.98 |
| Dyad intercept SD | 0.26 | 0.16 | 0.02, 0.60 |  | 0.18 | 0.13 | 0.01, 0.49 |  | 0.31 | 0.21 | 0.01, 0.78 |
| Dyad training SD | 0.23 | 0.15 | 0.01, 0.57 |  | 0.22 | 0.15 | 0.01, 0.56 |  | 0.53 | 0.18 | 0.13, 0.89 |
| Dyad intercept-training correlation | 0.43 | 0.53 | -0.85, 0.99 |  | 0.24 | 0.57 | -0.89, 0.98 |  | 0.19 | 0.53 | -0.90, 0.95 |
| Residual SD | 0.33 | 0.14 | 0.07, 0.55 |  | 0.55 | 0.13 | 0.21, 0.77 |  | 0.35 | 0.15 | 0.09, 0.64 |
| *Note*. 95% CI = 95% credibility interval; CSRS = CMH Session Reaction Scale; *M* est = mean of posterior distribution; *SD* = standard deviation of posterior distribution | | | | | | | | | | | |

References

Bürkner PC. brms: An R package for Bayesian multilevel models using Stan. Journal of Statistical Software 2017;80(1):1-28. doi:10.18637/jss.v080.i01

Cohen J. A coefficient of agreement for nominal scales. Educational and Psychological Measurement 1960;20(1):37-46.

Elliott R. The Revised Session Reaction Scale. Toledo, OH: University of Toledo; 1993.

Gelman A, Rubin DB. Inference from iterative simulation using multiple sequences. Statistical Science 1992;7(4):457-72. doi:10.1214/ss/1177011136

Neal RM. MCMC using Hamiltonian dynamics. In: Brooks S, Gelman A, Jones G, Meng X-L, editors. Handbook of Markov Chain Monte Carlo. Boca Raton, FL: CRC Press/Chapman & Hall; 2011. p. 113-162.

Rosseel, Y. (2012). lavaan: An R package for structural equation modeling. *Journal of Statistical Software, 48*(2), 1-36.

Rosseel Y. lavaan: An R package for structural equation modeling. Journal of Statistical Software 2012;48(2):1-36. Retrieved from http://www.jstatsoft.org/v48/i02/

The Stan C++ Library [computer program]. Version 2.14.0. Stan Development Team; 2016. Retrieved from http://mc-stan.org
