# Supplementary material for: Nonprofessional Peer Support to Improve Mental Health: Randomized Trial of a Scalable Web-Based Peer Counseling Course
Source: J Med Internet Res. 2020 Sep 21;22(9):e17164. doi: 10.2196/17164 (PMC7536598; doi:10.2196/17164)
Supplement: Multimedia Appendix 2 [file jmir_v22i9e17164_app2.docx]

**CMH SESSION REACTION SCALE**

| **1** | **2** | **3** | **4** | **5** | **6** | **7** | **8** | **9** |
| --- | --- | --- | --- | --- | --- | --- | --- | --- |
| **Not at all** |  | **Slightly** |  | **Somewhat** |  | **Pretty Much** |  | **Very Much** |

[Items were administered in a random order.]

1. As a result of this session, I have realized or become clearer about what I need or want to work on, or what my problems or goals are.

2. As a result of this session, I have come to understand myself, or my feelings, or my actions better.

3. As a result of this session, I now feel more supported by my partner.

4. As a result of this session, I feel closer to my partner; I have come to experience my partner as a person or fellow human being; or I feel less alone because of my relationship with my partner.

5. As a result of this session, I now feel understood by my partner; or I am glad that my partner really understood what I was thinking, feeling, or trying to say.

6. As a result of this session, I have become more aware of things about my situation or about other people in my life (not counting my partner); or I am facing the reality of a situation.

7. As a result of this session, I am now more in touch with my feelings or thoughts; I have realized something about myself; or I have become clearer about things in myself that I had been avoiding or having trouble putting into words.

8. As a result of this session, I am more accepting of who I am; or I have come to see myself or specific things about me more positively or less negatively.

9. As a result of this session, I have figured out how to go about resolving a specific problem or how to achieve a specific goal; or I decided what to do about my problems or situation.
